# Induced Mutagenesis and Comparative Genomics of *Raoultella* sp. 64 for Enhanced Antimony Resistance and Biosorption

**DOI:** 10.3390/microorganisms13040880

**Published:** 2025-04-11

**Authors:** Tianhua Huang, Shiran Cao, Xiaohan Li, Chuhan Wang, Xiawei Peng

**Affiliations:** 1 School of Biological Sciences and Technology, Beijing Forestry University, Beijing 100083, China; huangtianhua0411@163.com (T.H.); 13020569871@163.com (S.C.); lixiaohan202503@163.com (X.L.); m15397530272@163.com (C.W.); 2 Institute of Tree Development and Genome Editing, Beijing Forestry University, Beijing 100083, China; 3 Beijing Key Laboratory of Food Processing and Safety in Forestry, Beijing Forestry University, Beijing 100083, China

**Keywords:** *Raoultella ornithinolytica*, space breeding, Sb(III)-Oxidizing bacteria, antimony adsorption, differential functional genes

## Abstract

Antimony-resistant bacteria are potential natural resources for the bioremediation of mining soil pollution. A *Raoultella* sp. 64 strain was isolated from antimony-contaminated soil. To enhance its Sb resistance abilities, this strain was transported into space aboard the Shenzhou spacecraft for space breeding, resulting in a mutant strain, *Raoultella* sp. D9. The whole genomes of *Raoultella* sp. 64 and mutant strain *Raoultella* sp. D9 were sequenced, revealing the genomic information for the bacterium. Comparative genomic analysis was then carried out to identify differential functional genes. The adsorption conditions for Sb(III) were optimized and refined. Further, Fourier transform infrared spectroscopy (FTIR) was used to determine the adsorption of antimony. Results show that strain D9 exhibits a higher tolerance to Sb(III), and Sb resistance genes were identified in both *Raoultella* sp. 64 and D9. Analysis of the differential functional genes indicated that the increased copy number of *pls*X may lead to a higher lipid content in the cell membrane, thereby enhancing the cell’s resistance to heavy metals. Mutant strain D9 exhibited better biosorption capacity compared to strain 64. FTIR studies showed that key functional groups, including -OH, C-N, C-H, and C-O, are likely to have participated in Sb(III) biosorption. Further study of the differential functional genes could provide a basis for future research and the subsequent development of technologies for the remediation of Sb-contaminated sites.

## 1. Introduction

Antimony (Sb), a metalloid element belonging to group 15 of the periodic table, is often mobilized as a toxic contaminant during mining activities [1]. Sb compounds have been classified as priority pollutants by the United States Environmental Protection Agency (USEPA) and the EU and are also identified as key pollutants in China’s *12th Five-Year Plan for Comprehensive Prevention and Control of Heavy Metal Pollution* [2,3]. China holds the world’s largest antimony reserves and dominates global antimony mining, accounting for approximately 78% of production capacity [4]. Significant emissions of Sb may impose substantial health risks on local populations, with both non-carcinogenic and carcinogenic risk values across all exposure pathways in mining and smelting areas exceeding acceptable limits [5]. Sb exists in four oxidation states (−III, 0, +III, and +V), with the +III and +V states being the most common in the environment [6]. Inorganic compounds of Sb are more toxic than its organic counterparts, with Sb(III) compounds predicted to be approximately 10 times more toxic than Sb(V) oxo-anionic species [7]. The abiotic oxidation of Sb(III) proceeds at a slow rate under neutral conditions. However, to thrive in such harsh environments, numerous bacteria have evolved diverse metabolic strategies, such as oxidation-reduction and secondary mineralization, which allow them to effectively reduce various toxic metals. This highlights their significant potential for use in the bioremediation of contaminated environments [8].

Microbes’ interactions with various form of Sb involve complex biogeochemical processes. Understanding the detoxification mechanisms employed by microbes is crucial for advancing bacterial bioremediation [9]. Efflux of Sb(III), which prevents the accumulation of Sb(III) within cells, is a key defense mechanism employed by microbes to mitigate the stress and toxicity caused by Sb(III). The ArsB transport protein plays a crucial role in regulating Sb(III)/As(III) efflux [10] and is found widely in Sb-oxidizing bacteria. The Acr3p protein and its homologous YqcL protein in the As transport family encoded by the acr gene cluster (*acr*1, *acr*2, and *acr*3) can substitute the ArsB protein as the Sb(III)/As(III) efflux pump [11]. Microbial oxidation that converts the toxic Sb(III) to less-toxic Sb(V) improves the microbial tolerance for Sb [12] and the adaptability to the natural environment. A comprehensive Sb(III) oxidation mechanism of *A. tumefaciens* GW4 was proposed by Li [13]. The co-metabolic process involves intracellular enzymatic catalysis by Sb(III) oxidase AnoA and As oxidase AioAB, as well as oxidation by cellular H_2_O_2_. Additionally, the presence of As oxidase AioA can impact both the enzymatic reaction catalyzed by AnoA and the non-enzymatic reaction mediated by H_2_O_2_ [13]. In addition, many Sb-oxidizing bacteria also exhibit excellent adsorption. EPS is a natural barrier for biological adsorption in order to restrict and prevent metal ion mobility and prevent entry into the cell. The capacity of EPS for Sb adsorption was mainly because of the reduction of Sb(V) to Sb(III) by hemiacetal hydroxyl, anilino (-NH-), and phenolic hydroxyl (-OH), accompanied by chelation [14]. However, in addition to the adsorption of Sb by EPS, the physical barrier provided by other components is also a crucial aspect of microbial tolerance to Sb. Chen et al. indicate that the adsorption of extracellular components, apart from EPS, can account for approximately 40% [9]. However, research on the mechanisms underlying this aspect has yet to achieve significant breakthroughs.

Microbial breeding through genome alteration plays a crucial role in biotechnological research and the bioindustry [15]. Mutagenesis breeding-induced mutations in the cell membrane have been shown to improve microbial resistance to extreme environments. Early studies from the 1970s revealed that nitrosoguanidine induces alterations in the phospholipid composition of *Escherichia coli* cell membranes, which in turn enhances the bacterium’s thermal tolerance [16]. Recent studies have demonstrated that mutations induced by atmospheric pressure room temperature plasma (ARTP) can alter the ratio of saturated to unsaturated fatty acids in the cell membrane. This modification in membrane composition enhances the acid tolerance of *Lactobacillus acidophilus* [17]. Mutation breeding not only improves strain performance and optimizes germplasm resources but also helps identify genetic variations underlying phenotypic changes, revealing potential new mechanisms. Zhao et al. screened for PQQ-producing bacteria and applied various mutagenesis methods to enhance PQQ yield. Whole-genome analysis of high-yield mutants was conducted to provide sequence data for exploring the molecular mechanisms of PQQ synthesis and supporting the molecular breeding of Methylobacterium [18].

During the previous phase of our study, we successfully isolated *Raoultella* sp. 64, an Sb(III)-tolerant strain, from slag samples collected in the Xikuangshan mining area. This strain can withstand Sb concentrations up to 3400 mg·L^−1^, while demonstrating an Sb(III) oxidation efficiency of 20.38%. Beyond the ozone layer, space gradually loses gravity, magnetic field, air, and environmental pressure [19]. We hypothesize that exposure to cosmic radiation during spaceflight may induce mutagenesis in strain 64, generating mutant strains with enhanced Sb(III) tolerance and adsorption capacity, and that comparative genomics would reveal key mutations in Sb resistance-related genes in order to reveal potential mechanisms.

In this study, we will screen for mutant strains with enhanced Sb tolerance through a comprehensive screening process. The minimum inhibitory concentration (MIC) of Sb(III) was preliminarily determined by assessing the growth response of strain 64 and its mutant derivatives at various Sb(III) concentrations. Next, whole-genome sequencing of both the wild-type strain 64 and its mutant derivative was performed, which revealed the genomic information fir the bacterium. Through systematic annotation, numerous key functional genes were identified. Comparative genomics analysis was performed to reveal the molecular adaptation mechanisms under Sb stress, and unearth the genetic differences behind these phenotypic changes. Furthermore, we characterized the adsorption profiles of both strains, elucidating the contributions of various cellular components to the Sb adsorption process.

## 2. Materials and Methods

### 2.1. Screening of Sb-Resistant Bacteria Mutant

Mutant strains of *Raoultella* sp. with increased resistance to Sb were selected as follows: bacterial cultures were inoculated into a modified CDM medium containing varying concentrations of Sb(III) and incubated at 28 °C for 16 h [20]. The minimum inhibitory concentration (MIC) of Sb(III) was determined by the absence of bacterial growth in the medium. After incubation, 1 mL of culture was withdrawn, centrifuged at 10,000× *g* for 2 min, and washed with saline. The cells were then serially diluted, plated on TSB agar (TSB, Oxoid, Basingstoke, UK), and incubated at 28 °C for 48 h. Single colonies that grew well were selected using inoculating needles and preserved (Figure S1). One colony, designated D9, with optimal growth, was selected for subsequent experiments.

### 2.2. Whole Genome Sequencing, Assembly, and Annotation

*Raoultella* sp. 64 and its mutant strain *Raoultella* sp. D9 were inoculated into TSB medium and incubated overnight at 28 °C conditions. Qubit (Thermo Fisher Scientific, Waltham, MA, USA) was used for precise quantification of DNA concentration, facilitating DNA detection. The genomic DNA was fragmented using a 26 G needle, and the fragments longer than 20 kb selected using BluePippin. Following this, end repair and A-tailing were performed, and adapters ligated to both ends of the fragments to prepare the DNA library. After ensuring that the library passed quality control, sequencing was conducted on the PacBio Sequel platform based on the effective concentration of the library and data output requirements. Raw data quality control was performed to filter out low-quality reads, retaining only high-quality reads; this filtered data was referred to as Clean Data. Subsequently, the genome was assembled to obtain sequence information that reflects the genomic makeup of the sample. Based on the assembled genome, coding regions were annotated with glimmer3 [21], tRNA with tRNAscan-SE V1.4 (http://lowelab.ucsc.edu/tRNAscan-SE/ accessed on 18 March 2024) [22], rRNA with rRNAmmer V1.2 (http://www.cbs.dtu.dk/services/RNAmmer/ accessed on 18 March 2024) [23], and sRNA with cmsearch and Rfam [24]. The predicted coding sequences were associated with Cluster of Orthologous Genes (COG) categories, Gene Ontology (GO) terms, Kyoto Encyclopedia of Genes and Genomes (KEGG) pathway terms, and eggNOG annotations using eggNOG-mapper version 0.99.2-3-g41823b2 [25]. Heavy metal(loid)s resistance genes in the tested genomic sequences were annotated with default parameters in the Biocide and Metal Resistance Gene Database (BacMet) (http://bacmet.biomedicine.gu.se accessed on 22 March 2024) [26].

### 2.3. Screening and Analysis of Differential Functional Genes

Comparative analysis was performed using NUCmer version 3.1 software, while SNPs and indels are detected using MUMmer version 4.0.0 software. The reference genome used for this analysis was *Raoultella* sp. 64, and the target genome D9. Following the detection of SNPs, gene annotations were applied to provide functional information related to the identified variants [27].

### 2.4. Pan-Genome Analyses of Raoultella *spp*.

Orthologs or orthologous genes are clusters of genes in different species that originated by vertical descent from a single gene in the last common ancestor [28]. In this study, we use OrthoVenn (https://orthovenn3.bioinfotoolkits.net/start/db accessed on 4 June 2024) for the comparison and analysis of genome wide orthologous clusters across multiple species. Four *Raoultella* species genome sequences were downloaded from NCBI; the accession numbers were GCA_006539725.1 (*Raoultella terrigena*) GCA_030389145.1 (*Raoultella terrigena*) GCA_022637595.1 (*Raoultella planticola*) and GCA_012029655.1 (*Raoultella terrigena*). The protein sequences were imported for analysis, with parameter settings of E-value = 1 × 10^−5^, Inflation value = 1.5. Homologous and unique genes between strain 64 and the other four genomes were investigated.

### 2.5. Sb(III) Sorption of Sb-Resistant Bacteria R. ornithinolytica

The effects of different conditions (initial concentration, adsorption time, and pH) on the adsorption of Sb(III) for the bacterial strain were investigated. Briefly, *Raoultella* sp. 64 and its mutant strain *Raoultella* sp. D9 were inoculated into TSB medium and incubated overnight at 28 °C conditions. The cultures were then aliquoted into 20 mL portions for subsequent experiments.

To investigate the optimal pH, the medium was treated with Sb(III), and the pH was adjusted to 5.5, 6.5, 7.5, 8.5, and 9.5. To determine the optimal initial concentration, different amounts of Sb(III) were added to achieve concentrations of 2.0, 4.0, 6.0, 8.0, 10.0, 15.0, and 20.0 mg·L^−1^, To identify the optimal adsorption time, the medium was treated with Sb(III) and incubated for 0, 30, 60, 90, 120, 150, and 180 min. Other factors were held constant at typical values (pH 6.5, 120 min, 6 mg L^−1^ Sb) while one factor was varied. All the experiments were performed in triplicate. (Table S1, Figure S2).

Next, the mixture was centrifuged at 8000× *g* for 5 min, and the supernatant was pipetted from the top layer, filtered through a 0.22 μm membrane filter and analyzed for Sb using Inductively Coupled Plasma–Optical Emission Spectroscopy(Agilent 5110 ICP-OES, Santa Clara, CA, USA). Adsorption rate was calculated using standard formula (Equation (1)):

(1) $$q=\frac{\rho_{0}-\rho_{e}}{\rho_{0}}\times 100\%$$

where *q* (%) = adsorption rate, *ρ*₀ (mg L^−1^) = initial concentration, and *ρₑ* (mg L^−1^) = equilibrium concentration.

The biosorption of Sb in cell fractions, including EPS and cell (without EPS) adsorption, was quantified [29]. The medium was treated with 6 mg·L^−1^ Sb(III) and incubated for 180 min. The supernatant-1 was collected by centrifugation at 8000× *g* for 5 min and analyzed for total Sb using ICP–OES. Precipitates-1 were collected and washed again with 0.05 mM Tri-HCl buffer solution at pH 7.0 three times. Then, supernatant-2 (Tri-HCl + EPS) was collected by centrifugation at 12,000× *g* for 15 min and diluted to the original volume to analyze the sorption capacity of EPS to Sb [30]. Next, the precipitate-2 was resuspended and broken by an ultrasonic crusher (150 W, operating for 5 s and intermittent for 5 s) [31]. The Supernatant-3 (cytoplasm) was collected by centrifugation at 12,000× *g* for 15 min; precipitate-3 consists of the cell walls and the cell membrane [32] (Figure S3).

### 2.6. FTIR and SEM–EDX Analysis

Both strains were incubated in CDM liquid medium with or without 600 mg·L^−1^ Sb(III). Cell powder were mixed with spectral grade KBr for FTIR analysis (Thermo Fisher Nicolet iS20 FTIR, Waltham, MA, USA). Spectral bands were smoothed and labeled (Thermo Scientific OMNIC Scientific, 8.0). The cell morphology was observed using a scanning electron microscope (FEI Nova Nano 450, Hillsboro, OR, USA).

### 2.7. Data and Statistical Analyses

Data were analyzed using IBM SPSS Statistics for Windows, Version 22.0 (IBM Corp, Armonk, NY, USA). Differences between groups were analyzed using the one-way ANOVA method and were considered significant at *p* ≤ 0.05.

## 3. Results

### 3.1. Screening of Sb(III) Tolerant Strains

Antimony-oxidizing bacteria, which play a pivotal role in environmental bioremediation and self-detoxification processes, possess the unique capability to transform toxic Sb(III) and As(III) into their less toxic pentavalent forms Sb(V). These microorganisms are widely distributed in nature and exist across various species. The Sb(III)-tolerant *Raoultella* sp. 64 strain was sent into space aboard the Shenzhou XIV spacecraft, which placed in a biological sample package designed specifically to meet the flight requirements. The screening work started immediately after the spacecraft returned.

**Figure 1:**
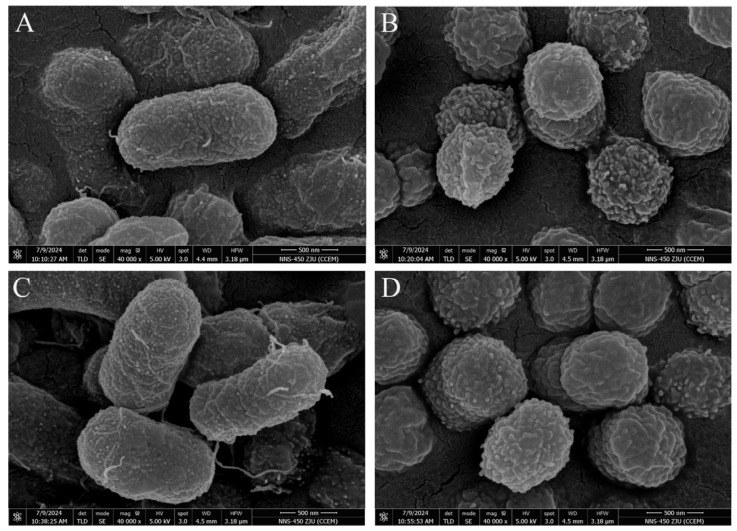
Cell morphology of *Raoultella* sp. 64 and D9 observed by scanning electron microscope (SEM). (**A**) SEM image of 64 incubation in CDM liquid medium. (**B**) SEM image of 64 incubation in CDM liquid medium under 600 mg·L^−1^ Sb(III) stress. (**C**) SEM image of D9 incubation in CDM liquid medium. (**D**) SEM image of D9 incubation in CDM liquid medium under 600 mg·L^−1^ Sb(III) stress.

Six colonies of strain 64, named D3, E3, D12, A12, A1, and D9, were selected through a screening process based on their tolerance to Sb concentrations ranging from 3400 to 4000 mg·L^−1^. All screened strains exhibited similar colony morphology characteristics, appearing smooth, moist, and light yellow in color. Among these, strain D9 demonstrated superior growth performance and was consequently chosen for subsequent experimental investigations. Morphological characterization revealed that strain D9 is a Gram-negative bacterium with rod-shaped cells measuring 1.2 µm in length (Figure 1). When subjected to Sb(III) stress at a concentration of 600 mg·L^−1^, the bacterial cells underwent morphological transformation, becoming spherical with a rough surface.

A quantitative comparison between the mutant and the original strain revealed that D9 exhibited higher Sb(III) tolerance, with an MIC value of 3800 mg·L^−1^, 11% higher than that of strain 64 (Table S2). The strain’s high resistance to Sb(III) indicates its strong detoxification ability, making it a promising candidate for Sb pollution remediation. The growth curve, measured by optical density (OD) at 600 nm, showed typical growth phases. After a 5-h lag phase with minimal OD change, the culture entered a rapid exponential growth phase and reached the stationary phase at around 15 h (Figure S3).

### 3.2. Genomic Analysis of 64

**Figure 2:**
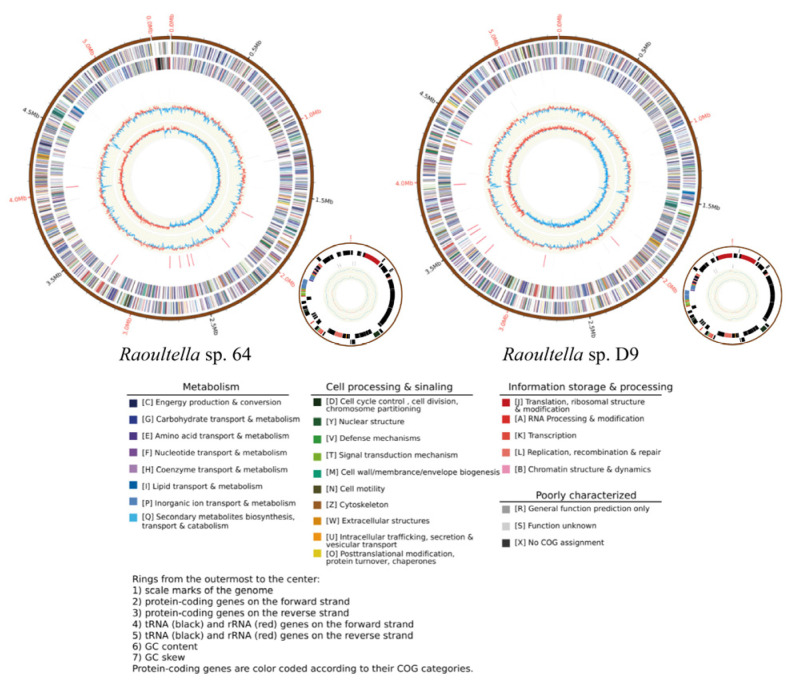
The circular genome maps of *Raoultella* sp. 64/D9 chromosome and plasmid. Corresponding to the rings from the outside to inside are genome, protein-coding genes (sense strand), protein-coding genes (antisense strand), tRNA and rRNA coding genes (sense strand), tRNA and rRNA coding genes (antisense strand), GC content, and GC skew.

To further understand the genetic mechanism for Sb(III) tolerance by strain 64, the whole genome of 64 was sequenced. The genome of *Raoultella* sp. 64 includes a single 5.3 Mbp chromosome and one plasmid (Figure 2). The circular chromosome consists of 5,351,241 bp with a G + C content of 55.91%. The chromosome contains 4975 coding sequences (CDS), 7 gene islands (GI), 89 tRNAs, and 25 rRNAs (Table 1). According to calculations from the FastANI (https://github.com/ParBLiSS/FastANI accessed on 20 March 2024), the ANI values of 64 with *Raoultella ornithinolytica* NCTC9164 exceeded the 98% threshold. Therefore, 64 was assigned to the *Raoultella* genus, ornithinolytica species and designated as *R. ornithinolytica* 64. Besides, we also analyzed the orthologous groups of proteins (COG) (Figure S3), the gene ontology (GO) (Figure S4), KEGG (Figure S5), and carbohydrate-active enzymes (CAZyme) of 64, These data suggest that the activity of 64 is mainly concentrated in catalysis and transport.

**Figure 3:**
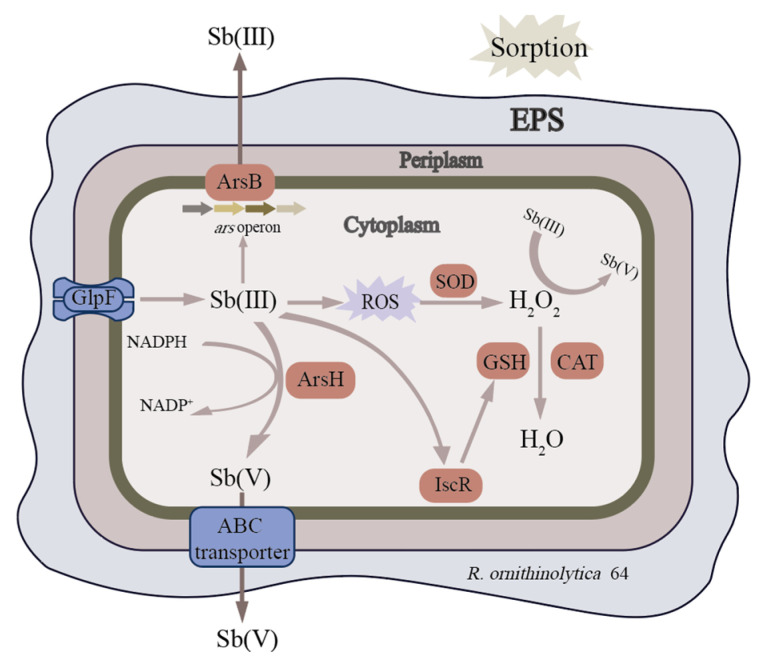
A schematic diagram of antimony resistance and oxidation of *Raoultella* sp. 64. Ovals represent enzymes that play a key role. The arrows indicate the transformation of substances or the action of different enzymes.

Potential Sb(III) resistance genes were identified in both strains and these genes can be classified into two categories: Sb efflux and Sb non-enzymatic oxidation. The *ars* operons comprise a set of ion transport genes, including the regulatory gene *ars*R, the arsenic effector genes *ars*A and *ars*B, as well as the auxiliary genes *ars*D and *ars*C that support the function of the former two effectors. Sb non-enzymatic oxidation refers to the process of Sb(III) oxidation mediated by reactive oxygen species (ROS) generated under cellular stress conditions. In strain D9, the ROD transformation-related genes *sod*B and *sod*C, the hydrogen peroxides catalytic gene *kat*E, and the glutathione synthesis regulatory gene *isc*R were identified. Additionally, this strain exhibits the *ars*H gene, which encodes an arsenate resistance protein and may be associated with both arsenic and Sb resistance (Figure 3).

Genomic annotation revealed the presence of multiple metal resistance genes, involved in the detoxification of Cu^2^⁺ (*cop*A), nickel transport (*nik* operon), and zinc uptake (*znu*ABC transporter system) (Table S2). This suggests that the genomes of *R. ornithinolytica* 64 and its mutant strain *R. ornithinolytica* D9 contain the molecular basis for resistance to Sb(III) and other heavy metals, which may contribute to the strains’ adaptive potential in mining environments, where multiple metal contaminants typically coexist.

### 3.3. Comparative Genomic of Raoultella

**Figure 4:**
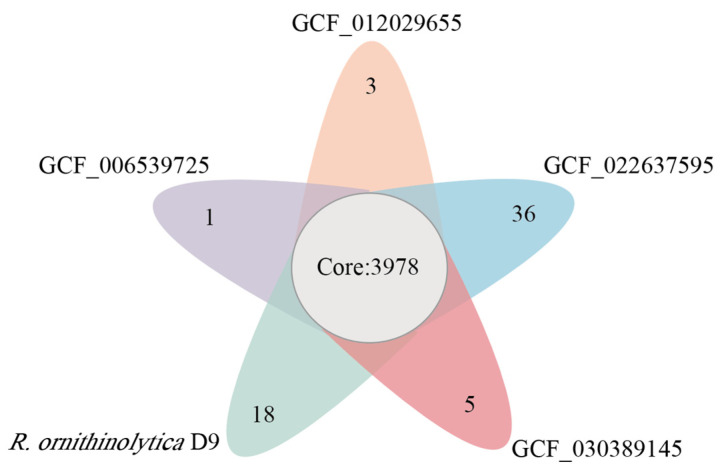
Cluster of orthologous group analysis of *Raoultella* sp. 64 and other 4 strains of *Raoultella*. Venn diagram of core, accessory, and unique genes.

To explore the orthologs and unique genes among *R. ornithinolytica* 64 and the other four genomes, genome-wide analysis of orthologous clusters was performed. A total of 5446 orthologous groups were clustered between strain 64 and the other four genomes. Notably, most 3978 OGs were shared by all four genomes, forming core groups that were conserved among the five strains. A total of 5110 (90.76%) genes of 64 clustered into 4638 OGs. Similarly, most of the genes in the other three genomes were included in the OGs: 5182 (93.77%, 4859 OGs), 5383 (86.42%, 4652 OGs), 5259 (92.17%, 4847 OGs) and 5437 (89.61%, 4872 OGs) genes for *R. terrigena* NBRC 14941, *R. planticola* A2-F21, *R. terrigena* JH01, and *R. terrigena* NXT28 were clustered into OGs, respectively (Table S3) (Figure 4). A total of 18 OGs were specific to strain 64, most of which were unannotated based on database comparisons. GO enrichment analysis of these specific OGs revealed associations with the response to arsenic-containing substances, suggesting that the strain may possess the ability to resist arsenic (Table S4). Future studies could employ targeted genetic manipulations to definitively these unique Ogs’ causality.

Through analysis of differential functional genes, we identified genetic variations in mutant strain D9, including one single nucleotide polymorphism (SNP), seven insertion fragment (Ins), and one deletion fragment (Del). The presence of three Ins may be associated with functional genes, specifically *pls*X and Polyphosphate: ADP phosphotransferase 3 (Table S5).

### 3.4. Sorption of Sb(III) by R. ornithinolytica 64 and D9

**Figure 5:**
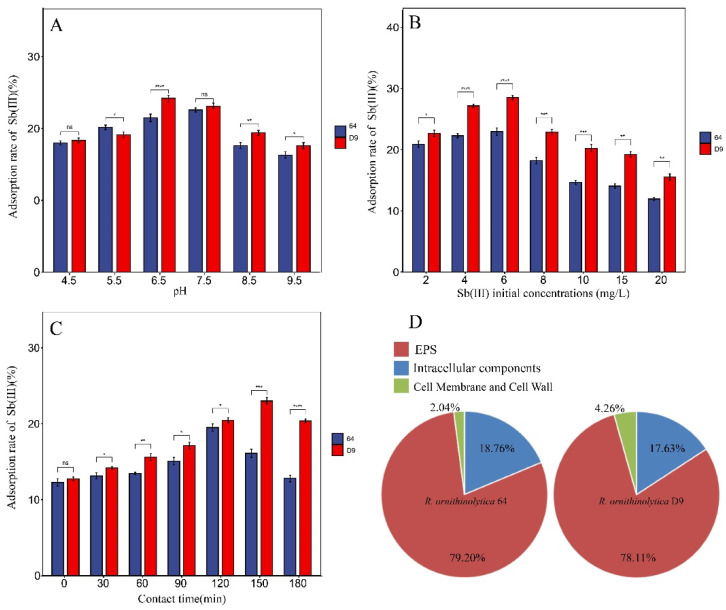
The effects of different conditions and different components on the adsorption of Sb(III) by both strains. (**A**) different pH, (**B**) Sb(III) initial concentration, (**C**) adsorption time, (**D**) different components. * Denotes significant difference, significance levels: * *p* < 0.05, ** *p* < 0.01, *** *p* < 0.001, **** *p* < 0.0001, ns: non-significant statistically.

The data presented for sorption of Sb(III) in different conditions are as follows: in the time gradient experiment, the removal rates of Sb(III) in wastewater by strain 64 were 12.27%, 13.13%, 13.44%, 15.08%, 19.50%, 16.10%, and 12.83%, respectively. For strain D9, the removal rates were 12.4%, 14.20%, 15.61%, 17,15%, 20.47%, 23.00%, and 20.39%, respectively. As the adsorption time increased from 0 to 90 min, the strain’s ability to adsorb the heavy metal Sb improved, reaching the highest adsorption rate at 120 min. However, when the adsorption time exceeded 120 min, the rate began to decline (Figure 5A). In the pH gradient experiment, the removal rates of Sb(III) in wastewater by strain 64 were 18.03%, 20.05%, 21.56%, 22.65%, 17.69%, and 16.33%, respectively. For strain D9, the removal rates were 18.41%, 19.20%, 24.28%, 23.14%, 19.47%, and 17.67%, respectively. As the pH of wastewater increased from 4.5–6.5, the efficiency of the strains in adsorbing the heavy metal Sb improved, reaching a maximum adsorption rate at 6.5. However, when the pH exceeded 8.5, the adsorption rate began to decline (Figure 5B). At different initial concentrations, the removal rates of Sb(III) in wastewater by strain 64 were 20.7%, 22.09%, 22.77%, 18.06%, 14.51%, 13.93%, and 11.84%, respectively. For strain D9, the removal rates were 22.49%, 26.95%, 28.26%, 22.69%, 19.47%, 17.67%, and 15.40%, respectively. As the initial concentrations of Sb(III) increased from 0–6 mg·L^−1^, the efficiency of the strains in adsorbing the heavy metal Sb improved, reaching a maximum adsorption rate at 6 mg·L^−1^. However, when the initial concentrations of Sb(III) exceeded 8 mg·L^−1^, the adsorption rate began to decline (Figure 5C). Results revealed that the mutant strain D9 demonstrated a significantly enhanced adsorption capacity (*p* < 0.05) compared to its wild-type counterpart, strain 64. The analysis revealed the distribution of cellular adsorption of Sb(III) as follows: for strain 64 with EPS, 79.20% of the Sb(III) was associated with EPS, 18.70% was in intracellular components, and 2.04% was in the cell walls without EPS. For strain 64 without EPS, 78.11% was in EPS, 15.45% in intracellular components, and 4.26% in the cell walls (Figure 5D). The EPS components were responsible for capturing the majority of Sb(III), demonstrating their strong retention capacity and effectively limiting the amount of Sb(III) that entered the cell.

**Figure 6:**
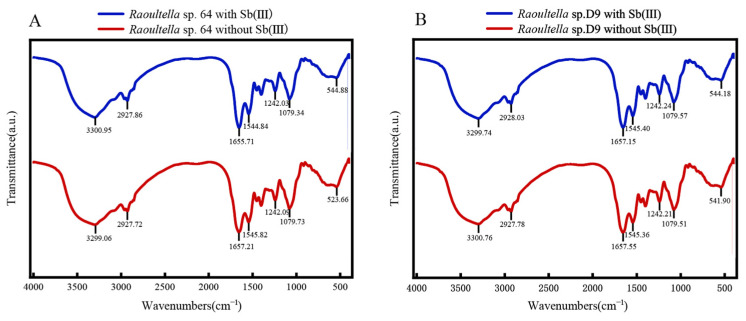
FTIR spectra of both strains. (**A**) *Raoultella* sp. 64 (**B**) *Raoultella* sp. D9.

To investigate the molecular mechanisms underlying Sb adsorption, Fourier-transform infrared spectroscopy (FTIR) was used to characterize the functional groups and identify binding components in strain 64 and D9. The FTIR spectrum of lyophilized microbes is shown in Figure 6. The spectrum reveals prominent bands between 3750 and 3000 cm^−1^, which correspond to the combined effects of hydroxyl (-OH) groups and amine and amide groups present in the amino acids. The bands between 3000 and 2700 cm^−1^ are linked to C-H stretching vibrations, particularly in alkanes (hydrocarbons). The peak between 1680 and 1630 cm^−1^ is typically associated with C=O stretching vibrations, common in ketones, aldehydes, esters, and other carbonyl-containing compounds. Bands between 1200 and 1500 cm^−1^ may correspond to COO, CCH, CC, CO, COH, and CN groups, primarily found in sugars, amino acids, proteins, amides, and humic substances. A peak near 542 cm^−1^ is attributed to OH and COH groups, commonly seen in sugars and proteins.

## 4. Discussion

Whole genome sequencing is convenient for understanding the antimony-promoting mechanisms of strains at the molecular level. The strain was identified as *Raoultella ornithinolytica*. From the perspective of genus level, Sb(III)-oxidizing bacteria mostly belong to *Pseudomonas*, *Comamonas*, *Acinetobacter*, *Rhizobium* and *Stenotrophomonas* [33]. In this study, the isolated strain *Raoultella* sp. 64 is the first strain of the genus *Raoultella* reported as having Sb oxidation ability. The annotation results of Sb resistance genes suggest that *R. ornithinolytica* strains 64 and D9 can expel Sb(III) through an arsenic efflux pump, which involves the ArsB transporter protein encoded by the *ars*B gene from the ion transport superfamily, as well as the enzyme encoded by the *ars*A gene [34,35]. In addition, Sb(III) can induce strains 64 and D9 to produce H_2_O_2,_ thereby oxidizing Sb(III) to Sb(V) to reduce its toxicity [36], and catalase *kat*E can catalyze excess H_2_O_2_ [37]. Sb(III) can also induce the expression of IscR and promote glutathione(GSH) synthesis, thereby depleting excess H_2_O_2_ and maintaining intracellular REDOX balance. Similar mechanisms may occur in arsenic-resistant bacteria, which further demonstrates this strain’s fundamental resistance to arsenic too. There is no definite conclusion on the role of *ars*H gene. Páez et al. believe that ArsH may alleviate the toxicity of arsenic species by mediating the reduction of reactive oxygen species (ROS) produced in cells when exposed to anions [38]. The *aio*AB and *ano*A genes involved in Sb(III) oxidation were not annotated in the genomes, but the strains still showed a high oxidation rate to Sb(III), which was consistent with the findings of Xiao et al. [39]. It is suggested that the oxidation process of Sb(III) may be regulated by multiple genes, or there may be an undiscovered oxidation pathway.

*R. ornithinolytica* 64 was subjected to mutagenesis through exposure to cosmic radiation and microgravity conditions during spaceflight, leading to the generation of a mutant strain, named D9. This mutant exhibited significantly enhanced resistance capabilities against heavy metal stress compared to the parental strain. In this study, the growth media used for *R. ornithinolytica* 64 (either MRS agar or diluted glycerol) did not apply any specific environmental selection pressure. Therefore, it can be concluded that the mutations observed in the mutant strains were solely induced by the space environment. To unearthing the genetic differences behind these phenotypic changes, a comparative genomic analysis was performed. *pls*X is a key enzyme in Gram-positive bacteria, coordinating the production of fatty acids and membrane phospholipids. In the phosphatidic acid biosynthetic pathway, it converts acyl-ACP to acyl-phospholipids. Studies have indicated that the deletion of *pls*X not only influences the fatty acid biosynthesis pathway in Streptococcus but has an impact on its acid adaptation response [40]. The overexpression of *pls*X significantly raises the lipid content, with saturated fatty acids, particularly palmitic acid (16:0), significantly increased, while unsaturated fatty acids, such as linoleic acid (18:2) and α-linolenic acid (18:3), show a slight increase [41]. In previous research, *pls*X was affected by heavy metals at sub-inhibitory concentrations; copper (Cu) and cadmium (Cd) can both cause oxidative damage to membrane phospholipids and compromise membrane integrity [42]. Heavy metals can also disrupt the cell membrane by altering lipid biosynthesis or generating ROS, which impair organelle function, protein synthesis, and damage nucleic acids [43]. On the other hand, membrane lipids can chelate metal ions, study shows that phosphate and carbonyl regions of phospholipid membranes can also bound to the metal cations [44]. Therefore, it can be deduced that the increased copy number of *pls*X may lead to a higher lipid content in the cell membrane of *R. ornithinolytica* D9, thereby enhancing the cell’s resistance to heavy metals.

Absorption is considered to be an important mechanism of resistance to Sb and approximately 12% of the adsorption occurred initially, indicating the strain’s rapid surface adsorption capability. The maximum adsorption rate was achieved between 120 and 180 min, likely due to the strain reaching adsorption equilibrium. Extending the adsorption time resulted in precipitation-reduced removal efficiency. Therefore, a longer adsorption time does not necessarily enhance the removal rate. The strain exhibited low adsorption efficiency under acidic conditions (pH < 6.5), possibly due to the acidic environment being unfavorable for the strain’s survival. Biosorption gradually increased when increasing pH from 3.5 to 6.5. Kiran et al. suggested that, at pH levels above 5.0, more negatively charged functional groups, such as carboxyl, amine, and hydroxyl, are exposed, providing more attraction sites for positively charged ions and enhancing biosorption capacity [45]. However, under alkaline conditions (pH ≥ 8.5), the formation of insoluble precipitates was observed, possibly due to the chemical interaction between Sb(III) and hydroxyl ions (OH-) in the solution, which results in a decrease in the strain’s adsorption rate. As the initial concentration of Sb(III) exceeds 6 mg·L^−1^, the adsorption rate decreases. This can be attributed to the saturation of available surface adsorption sites on the bacterial cells. This adsorption pattern is consistent with the Pb(II) adsorption characteristics observed in bacterial strains, as previously reported by Li et al. [46]. Some studies suggest that the decreased metal removal efficiency is probably due to insufficient metal binding sites on biomass [47,48], or it may be attributed to the increased toxicity of cadmium concentration in cell growth [49,50]. While our single-factor experiments successfully explained the individual effects of pH, contact time, and initial concentration on Sb(III) biosorption and uncovered the potential adaptation mechanism, the real environment is variable. Therefore, future studies could conduct full-factorial experiments to test cross-combinations of different conditions. The EPS components were responsible for capturing the majority of Sb(III), demonstrating their strong retention capacity and effectively limiting the amount of Sb(III) that entered the cell. These findings are consistent with previous studies [51]. The genome sequence indicated that the presence of *glp*F, GlpF can transport Sb(III) into cells [52]. However, the retention rate of Sb in the cells is low; one of the reasons is that EPS can act as a protective barrier, while another is that *ars*B gene can encode the ArsB transport protein, which is a trivalent metal/HC antiporter. When ATP hydrolase ArsA exists, ArsB transport protein can efflux Sb(III)/As(III), with energy produced by hydrolysis of ATP [34]. The adsorption of the mutant strains on the cell wall and cell membrane was significantly increased. Metal ions can bind to phospholipids, and the increased lipid expression in the mutant strains may affect the bioavailability and toxicity of heavy metals. Studies on the adsorption mechanism of Sb(III) to both strains showed that hydroxy- and carbohydrate were the predominant functional groups for surficial Sb(III) binding. Sb(III) might form complexes with hydroxyl-, carboxyl-, and amino- through ligand exchange reactions, which could also contribute to the adsorption of Sb(III) to the functional groups on the cell surface [53].

While this study revealed the Sb(III) resistance and adsorption mechanisms of mutant strain D9, its practical application requires further validation through pot experiments. Future work will focus on evaluating the strain’s colonization stability, long-term remediation efficiency, and plant–microbe interactions in contaminated soil, providing critical data for field applications.

## 5. Conclusions

*Raoultella* sp. 64, an antimony-oxidizing bacterial strain, was originally isolated from slag sample in the Xikuangshan mining area. In this study, we generated and screened a mutant derivative, *Raoultella* sp. D9, which exhibited better Sb tolerance compared to the wild-type strain. Whole genome sequencing analysis revealed that *Raoultella* sp. 64 is the first documented instance within the *Raoultella* genus to prove both antimony resistance and oxidation capabilities. Both 64 and D9 confirmed the presence of genes related to arsenic oxidation and As/Sb resistance, with 64 exhibiting more heavy metal(loid) resistance genes and higher gene expression compared to other strains of the same genus. Analysis of differential functional genes shows that an increased copy number of *pls*X may lead to a higher lipid content in the cell membrane, thereby enhancing the cell’s resistance to heavy metals. The specific mechanism merits further study. Surficial functional groups in the EPS and on cell walls/membrane of *R. ornithinolytica* 64 and D9 can immobilize Sb(III), thereby decreasing extracellular Sb(III) concentrations and toxicity.

## Figures and Tables

**Table 1:** Functional features of *R. ornithinolytica* 64/D9.

Strains	*R. ornithinolytica* 64	*R. ornithinolytica* D9
Size (bp)	5,351,241	5,351,236
Total genes	4975	4974
Average length (bp)	947.81	948.13
GC%	55.91	55.91
tRNA	89	88
rRNA	25	25
Gene island	7	7

## Data Availability

The original contributions presented in this study are included in the article. Further inquiries can be directed to the corresponding author.

## Supplementary Materials

The following supporting information can be downloaded at: https://www.mdpi.com/article/10.3390/microorganisms13040880/s1, Figure S1. The schematic diagram of Sb-resistant bacteria mutant screening experiment. Figure. S2. The schematic diagram of different conditions experiment. Figure S3. The schematic diagram of different compounds sorption experiment. Figure S4. Growth of 64 and D9 in CDM medium. Figure S5. COG classification statistics for *Raoultella* sp. 64/D9 genome annotation. Figure S6. GO classification statistics of *Raoultella* sp. 64/D9 genome annotation. Figure S7. KEGG classification statistics of *Raoultella* sp. 64/D9 genome annotation. Table S1. Adsorption Experiments. Table S2. Minimum inhibition concentration of Sb(III) of two stains. Table S3. Heavy metal(loid)s resistance genes. Table S4. Information of five *Raoultella* spp. Table S5. GO enrichment analysis for the specific OGs. Table S6. Result of SNP and InDel.
