# Supplementary material for: Induced Mutagenesis and Comparative Genomics of Raoultella sp. 64 for Enhanced Antimony Resistance and Biosorption
Source: Microorganisms. 2025 Apr 11;13(4):880. doi: 10.3390/microorganisms13040880 (PMC12029485; doi:10.3390/microorganisms13040880)
Supplement: Supplementary file 1 [file microorganisms-13-00880-s001.zip › microorganisms-3563197-supplementary.pdf]

Supplementary Materials

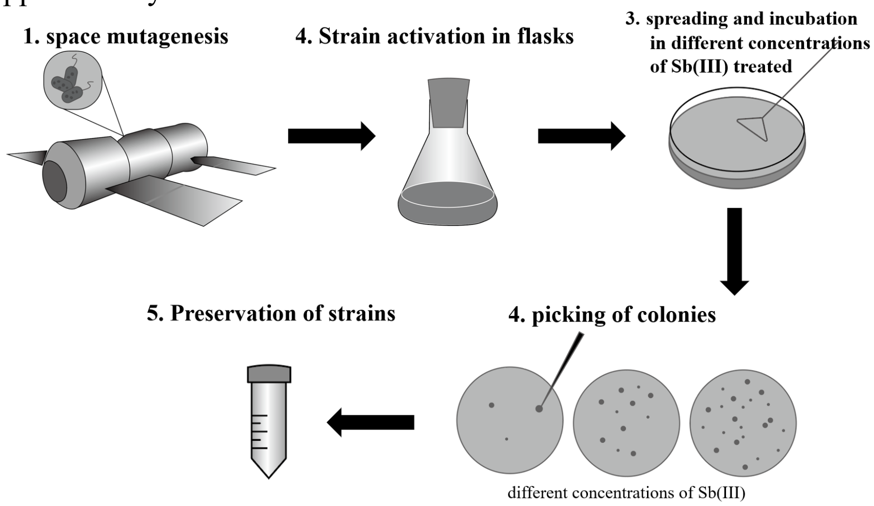

Figure S1. The schematic diagram of Sb-resistant bacteria mutant screening experiment

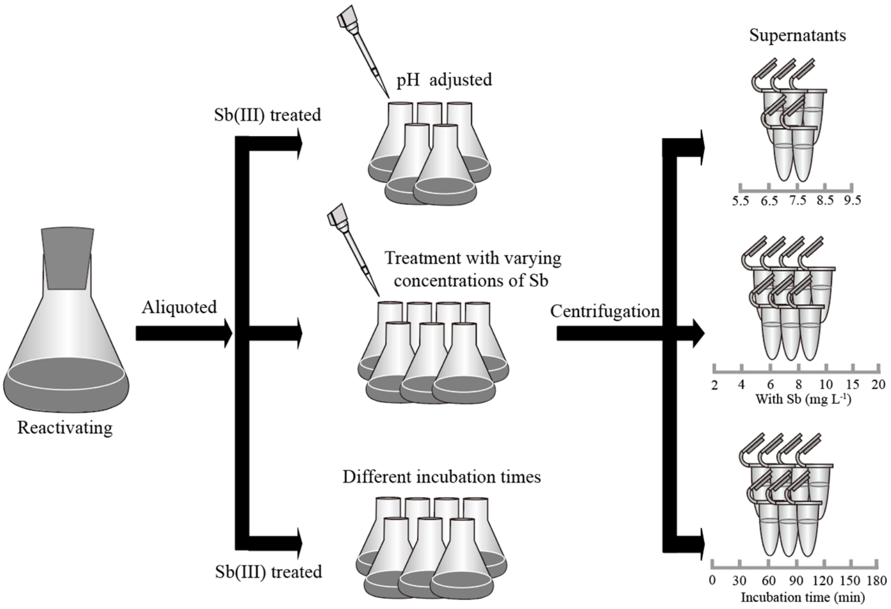

Figure S2. The schematic diagram of different conditions experiment

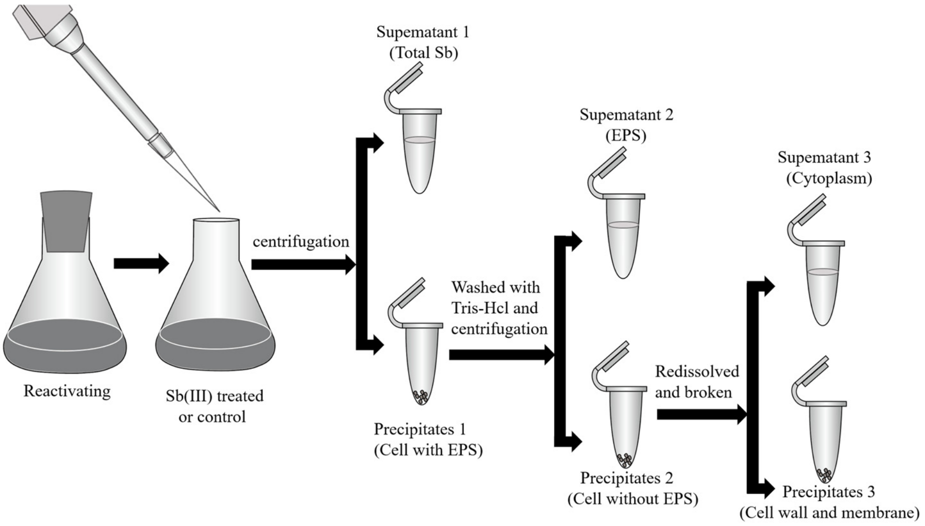

Figure S3. The schematic diagram of different compounds sorption experiment

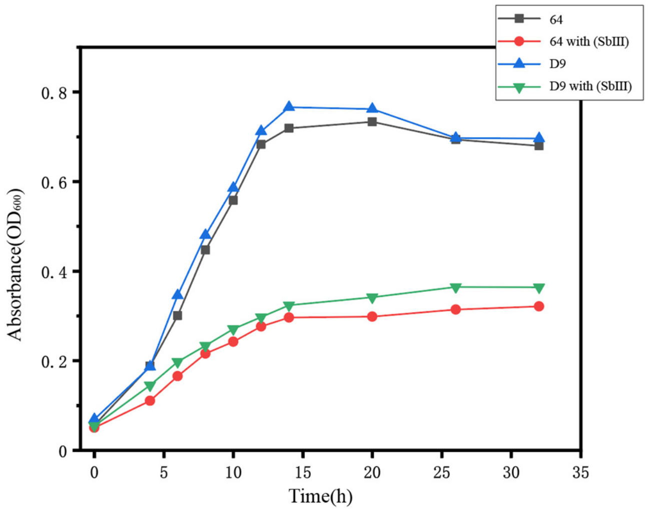

Figure S4. Growth of 64 and D9 in CDM medium

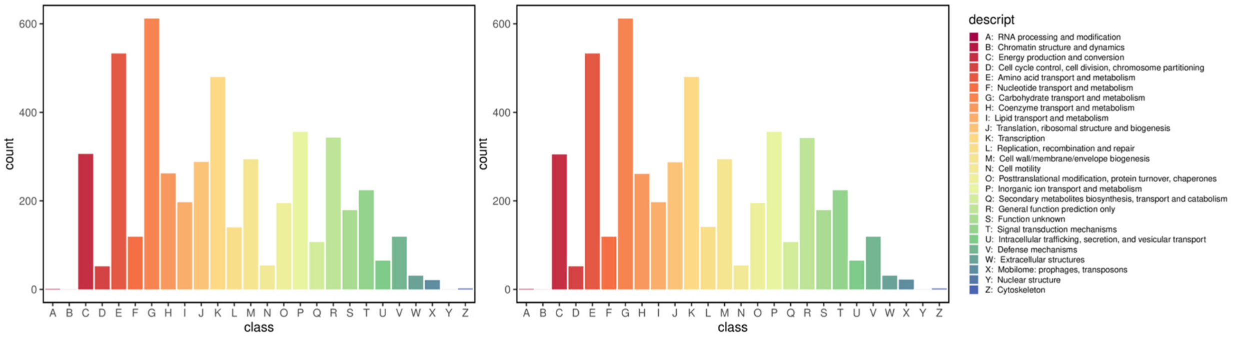

Figure S5. COG classification statistics of *Raoultella* sp. 64 / D9 genome annotation.

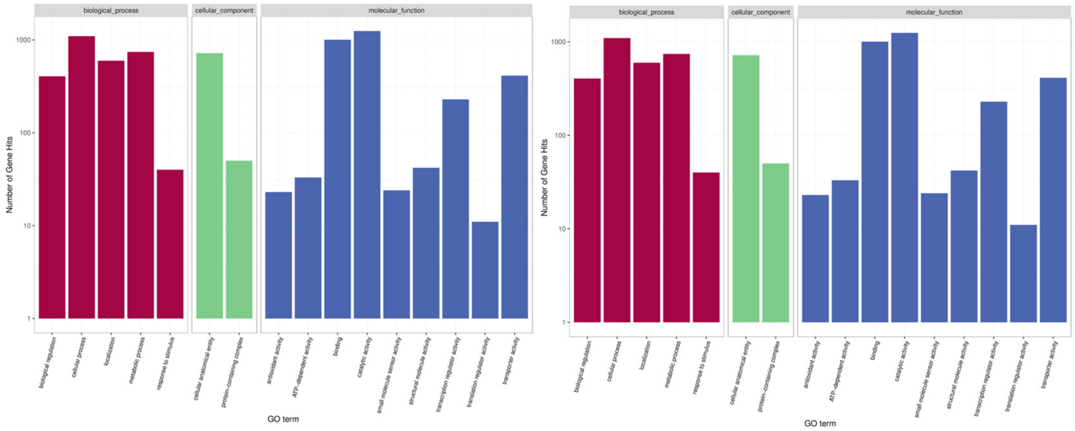

Raoultella sp. 64 *Raoultella* sp. D9

Figure S6. GO classification statistics of *Raoultella* sp. 64 / D9 genome annotation.

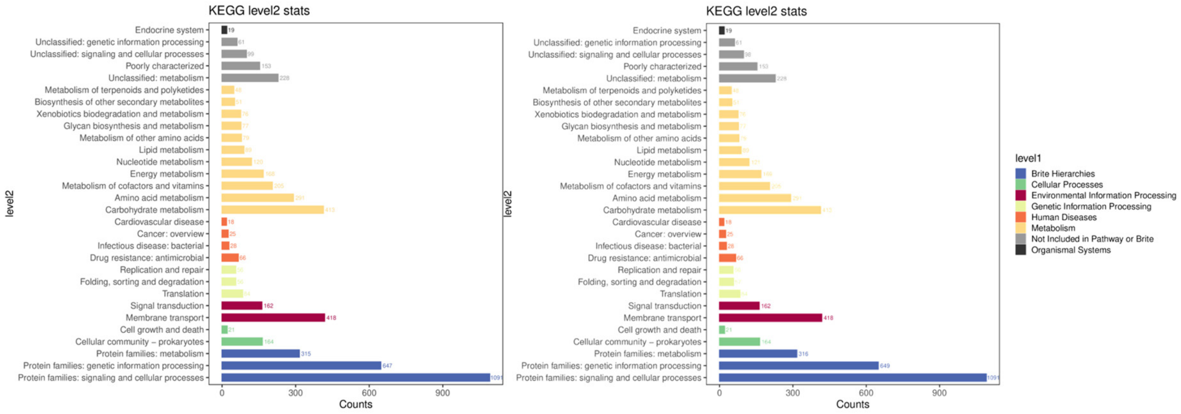

Raoultella sp. 64 *Raoultella* sp. D9

Figure S7. KEGG classification statistics of *Raoultella* sp. 64 / D9 genome annotation.

Table S1. Adsorption Experiments

Factor	Range						
pH	5.5	6.5	7.5	8.5	9.5	/	/
Sb(III) initial concentration (mg L^{-1})	2	4	6	8	10	15	20
Contact time (min)	0	30	60	90	120	150	180

Table S2. Minimum inhibition concentration of Sb(III) of two stains

**Strain name**	**Minimum inhibition concentration of Sb(III) (mg/L)**	**Gram stain**
64	3400	Gram-negative
D9	3800	Gram-negative

Table S3. Heavy metal(loid)s resistance genes

**Gene**	**Functions**
*ars*H	Arsenic (As) and Antimony (Sb) resistance-related
*ars*R	*ars* operon regulation
*ars*D	Assistance in arsenic (As) and antimony (Sb) efflux
*ars*A、*ars*B	Arsenic (As) and antimony (Sb) efflux
*ars*C	Arsenic (As) and antimony (Sb) reduction
*sod*B、*sod*C	ROD transformation
*kat*E	H_2O_2 catalysis
*isc*R	Glutathione (GSH) synthesis regulation
copA、*copS*、*cusB*、*cusC*	Copper (Cu) resistance
znuA、*znuB*、*znuC*	Zinc (Zn) resistance
nikA、*nikB*、*nikC*、*nikE*、*nikR*	Nickel (Ni) transport
sitA、*sitB*、*sitC*、*sitD*	Iron (Fe) resistance

Table S4. Information of five *Raoultella* spp.

Genome assembly	Taxon	genes	GC percent
/	*Raoultella ornithinolytica* 64	5110	55.8
ASM653972v1	*Raoultella terrigena*	5182	57.5
ASM3038914v1	*Raoultella terrigena*	5437	57.0
ASM2263759v1	*Raoultella planticola*	5383	55.5
ASM1202965v1	*Raoultella terrigena*	5259	57.5

Table S5. GO enrichment analysis on the specific OGs

GO ID	Name	Namespace	P-value
GO:0071722	detoxification of arsenic-containing substance	biological_process	0.00019
GO:0046685	response to arsenic-containing substance	biological_process	0.00219

Table S6. Result of SNP and inDel

Type	P1	SUB1	SUB2	P2	PRODUCT1	PRODUCT2
Ins	232149	.	G	232148	ADP phosphotransferase 2	ADP phosphotransferase 3
Ins	232150	.	C	232148	ADP phosphotransferase 2	ADP phosphotransferase 3
Ins	749576	.	T	749573	NonCoding	*pls*X
Ins	808505	.	A	808501	*rut*D	*rut*D
Ins	3132778	.	A	3132773	*puu*E	*puu*E
Del	3713214	T	.	3713210	Noncoding	Noncoding
Ins	4790236	.	C	4790231	*prc*	*prc*
Ins	4790237	.	T	4790231	*prc*	*prc*
SNP	3172894	T	G	3172889	*mal*T	*mal*T

[P1] position of the SNP in the reference sequence; [P2] position of the SNP in the query sequence; [SUB1] character or gap at this position in the reference; [SUB2] character or gap at this position in the query.
